# Evaluating the Cultural Adaptation of Evidence-Based HIV Prevention Interventions for African Immigrant Women: Exploratory Pilot Mixed-Methods Study

**DOI:** 10.1007/s10903-025-01762-2

**Published:** 2025-08-13

**Authors:** Nipher Malika, Laura M. Bogart, Nabila Adamu, Gray Maganga, Elaine D. Jeon, Esete Habtemariam Fenta, Khady Diouf, Bisola Ojikutu

**Affiliations:** 1 RAND Corporation, 1776 Main St, Santa Monica, CA 90401 USA; 2 Charles R. Drew University of Medicine and Science, Los Angeles, CA USA; 3 Brigham and Women’s Hospital, Boston, MA USA; 4 Harvard Medical School, Boston, MA USA

**Keywords:** African-born black women, HIV prevention, Condom use, PrEP use

## Abstract

African-born Black women living in the US experience markedly higher rates of HIV diagnosis than their US-born counterparts, with condom use and PrEP remaining underutilized despite their effectiveness. Existing HIV prevention interventions for African-born Black women are limited in scope; some lack cultural tailoring, linguistic appropriateness, and most not do include PrEP. Using the ADAPT-ITT model, we culturally adapted two evidence-based interventions for US Black women–Sister-to-Sister and Sisters Informing Sisters about Topics on AIDS (SISTA)–to increase condom use and PrEP uptake among African-born Black women through community stakeholder input. *DADA*, which was adapted from SISTA, consists of two 3-hour peer-led, group-level intervention and *Dada kwa Dada (DKD)*, adapted from Sister-to-Sister, is a 1-hour individual-level intervention. To test feasibility and acceptability, 29 African-born women without HIV were recruited from social media groups and community partner listservs in Massachusetts and New York; 17 were randomized to *DKD* and 12 to *DADA*. Participants completed risk assessments at baseline and provided post-intervention feedback interviews and surveys. Both adapted interventions demonstrated high feasibility and acceptability, with participants expressing positive qualitative and quantitative feedback regarding their culturally appropriateness, and relevance. This study addresses critical gaps in tailored HIV prevention approaches for African-born Black women and paves the way for future trials to improve condom use and PrEP in this population. Next steps are to conduct a fully-powered comparative effectiveness trial to assess the relative impact of both interventions on increased condom use and uptake of PrEP.

## Background

African-born Black women in the US face the highest rates of HIV diagnosis among Black individuals, with rates 5.3 times higher than US-born Black women and 1.4 times higher than US-born Black men [1–3]. Data suggest that a significant portion of HIV transmission within this diverse group occurs post-migration to the US which highlights the need for targeted prevention strategies within this immigrant community [4–8]. Research shows that HIV preventative behaviors such as uptake of condom use and PrEP use have been effective in reducing the risk of HIV transmission [9–11]; however, within African-born Black individuals in the US, uptake of such preventative behavior is limited [12].

Studies reveal cultural and linguistic barriers to engaging African-born Black individuals in discussing sexual health issues and uptake of HIV preventative behaviors [1, 2, 12–14]. Key cultural factors influencing African immigrants’ engagement in HIV prevention and risk behavior include misconceptions that wearing an amulet around the waist can protect against HIV or poor knowledge that HIV can be transmitted through mosquito bites [15–18]. Gender roles also play a crucial part; it is often frowned upon for women to discuss sex, and they frequently lack the power to negotiate safe sex with their partners, relying on male approval to use condoms [15, 16]. In cases of male infidelity, women are expected to be submissive and avoid discussing sexual health with their partners [1, 19]. Additionally, risk perception is skewed, with a belief that being in a monogamous relationship lowers HIV risk, even though partners may not be monogamous [12, 20, 21]. Women report having little power over other risk factors, such as gender-based power relations, economic insecurity, and domestic violence [1, 18, 22]. High HIV stigma further complicates the issue, as individuals with HIV face significant stigma, and even the process of getting tested can be stigmatizing, deterring many from seeking testing [12, 14]. Such cultural and linguistic barriers can impede the uptake of PrEP and condom use by preventing African-born women from understanding the benefits of condoms and PrEP for their sexual health. Furthermore, studies show that linguistic and cultural concordance are important for uptake of HIV related prevention services [14, 19, 23–25]. These factors highlight the necessity for culturally responsive interventions to address these challenges.

Efforts to develop culturally tailored HIV prevention interventions—those aligned with the cultural values, beliefs, norms, and practices of African-born Black women—and linguistically appropriate interventions—delivered in a language that is accessible and understandable—are extremely limited in this area. While the Safety Net Party is one such example, such interventions remain rare [19]. While existing HIV prevention interventions have proven effective for US born Black women [26–31], they may not adequately address the cultural nuances and barriers faced by African-born Black women living in the US [14]. Moreover, few of these interventions include the promotion of pre-exposure prophylaxis (PrEP).

Because culture plays a significant role in both amplifying and reducing risk for HIV [14, 32], in the present study, we identified two existing evidence-based interventions that could be culturally adapted for use by African-born Black women: Sister-to-Sister (S2S) [29] and Sisters Informing Sisters about Topics on AIDS (SISTA) [26–28]. S2S is a health profession-led individual-level intervention based on Social Cognitive Theory, and SISTA is a peer-led group-level intervention that addresses gender inequality using the Theory of Gender and Power. Both have been effective in increasing condom use and reducing sexuality transmitted infections among US-born Black women. We chose SISTA and S2S because they are evidence-based interventions that have demonstrated effectiveness in promoting safer sexual behaviors among U.S.-born Black women, utilize strengths-based and peer-led approaches, and align well with the values and social structures of African-born communities, making them suitable for culturally relevant adaptations. Adapting these interventions, rather than creating entirely new ones, offers several advantages. First, both S2S and SISTA have demonstrated effectiveness in promoting safer sexual behaviors, such as condom use, in U.S.-born Black women, providing a strong evidence-based foundation for adaptation. Leveraging these established frameworks allows researchers to focus on tailoring content to address the unique cultural, linguistic, and social needs of African-born Black women. Furthermore, adapting these existing evidence-based interventions was more time- and resource-efficient, enabling quicker implementation and testing in target populations. This approach also ensured that the proven strategies, such as peer-led discussions and community-based delivery models, were retained while incorporating culturally relevant modifications.

Guided by the ADAPT-ITT model [33] – a method for adapting evidence based HIV interventions – we engaged in formative research, conducting interviews and focus groups with African-born Black women and clinicians to culturally adapt these two evidence-based interventions, as well as to update them to promote PrEP use. We then conducted a mixed-methods comparative effectiveness pilot to assess feasibility by examining retention in intervention sessions, recruitment, and retention in follow-up assessments [34–37], while also evaluating the acceptability of the interventions. We hypothesized that the cultural adaptation of SISTA would be more acceptable than S2S because it addresses gender inequality – a primary driver of HIV infection – as cultural norms and power dynamics within relationships often restrict women’s roles and their ability to discuss sexual health [38, 39]. Additionally, SISTA’s group-level format fosters a sense of community and social connections, which is particularly valuable in collectivist cultural contexts [40, 41].

## Methods

### Study Setting and Community Partnerships

All study activities were conducted in the context of a community-academic partnership with African-born Black patients, clinicians, researchers, non-research African-born Black women, and various other stakeholders in the greater Boston area, MA and New York, NY. The research team is made up of majority African-born Black researchers and have a long history of working with African-born Black communities in the U.S. to provide support and address health disparities, which enhanced the cultural relevance of the study, fostered trust with community stakeholders and provided nuanced insights during data interpretation to ensure findings were contextually grounded. The main community partners included Multicultural AIDS Coalition (MAC) in MA and African Services Committee (ASC) in NY. MAC works to support HIV services, address health care access, homelessness, immigration challenges and discrimination. ASC is focused on expanding HIV prevention and access to care, as well as providing legal and other support services. In addition to ASC and MAC, we collaborated with other community partner organizations dedicated to serving African immigrant women populations including Whittier Street Health Center in Massachusetts and the Muslim Women’s Institute for Research and Development in New York. Partner organizations participated in weekly team meetings, advising on adaptation and inclusion strategies, implementation, advertising strategies, and establishment of effective and sustainable connections with these communities in their catchment areas.

This study was reviewed and approved by Massachusetts General Brigham institutional review board under approval numbers 2020P001558 and 2021P001486.

### Phase 1: Cultural Adaptation

#### Framework

Cultural adaptation was influenced by the ADAPT-ITT model [33], a method designed to facilitate the adaptation of existing evidence-based interventions in novel situations. The model is guided by eight steps outlined in Table 1 from assessment to testing. Our Assessment aimed to obtain a comprehensive understanding of African-born women through focus groups and interviews. Following this, Decision-making took place where evidence-based interventions (S2S and SISTA) were selected, and the decision to adapt was made. S2S and SISTA were strategically chosen because they demonstrated effectiveness in promoting safer sexual behaviors among U.S.-born Black women and had nationwide success. These interventions also addressed key needs identified by the community partners: promoting safer sex practices and HIV testing, empowering Black women to negotiate safer sex practices, and reducing HIV-related stigma within the community. Adaptation involved engaging stakeholders to culturally tailor S2S and SISTA, identifying which aspects of these interventions needed modification to meet the specific needs of the target population. For example, we identified the need to replace African American women’s poetry with African ethnic storytelling, changed basic health videos to be African-centric with African actors, and made the interventions bilingual in English and French. Once adaptation strategies were identified, the Production phase commenced, where S2S was adapted to Dada-kwa-Dada and SISTA to DADA. We also included assessments, HIV education including HIV transmission, condom use, and PrEP, and HIV Video featuring African-born women. Topical experts played a crucial role during this phase, providing substantive content and technical assistance. Integration followed, where all forms of information were combined to ensure coherence and effectiveness. Subsequently, Training was conducted to equip all relevant personnel with the necessary skills and knowledge. Finally, the process concluded with Theatre testing, and finally a pilot testing to assess the efficacy of the adapted interventions.

**Table 1:** Applying the ADAPT-ITT model to guide the adaptation S2S and SISTA

Phase	What we did
Assessment *Obtain a comprehensive understanding of the target population*	Conducted meetings with community stakeholders
Decision *Select interventions and decided whether to adopt or adapt*	Decided on two existing evidence-based interventions that could be culturally adapted for use by African-born Black women: Sister-to-Sister (S2S) and Sisters Informing Sisters about Topics on AIDS (SISTA)
Adaptation *Use a pretested methodology to better understand how to adapt*	Conducted virtual stakeholder meetings where general overview of S2S and SISTA was presented Themes interviews and focus groups were used to guide adaptation Retained core elements of S2 & SISTA: Communication with partner, condom use demonstration, sexual assertion skills Adaptation would involve the materials used, and the various activities within modules of S2S and SISTA
Production *Create an adaptation plan and determine goals*	Designed 2 adapted versions: Dada-kwa-Dada (DKD; the adapted version of S2S) and DADA (the adapted version of SISTA) Included assessments, HIV education including HIV transmission, condom use, and PrEP, and HIV Video featuring African-born women
Topical Experts *Provide substantive content and technical assistance*	Adapted interventions were reviewed by stakeholders, and clinical experts
Integration *Integrate all forms of information*	All forms of information (intervention protocols, videos, assessments, educational powerpoints) were combined to ensure coherence and effectiveness
Training *Train all relevant personnel*	All personnel were trained
Testing *Theatre test and Pilot test for adaptation efficacy*	A convenience sample of 29 women (18 English-speaking, 11 French-speaking) participated in the pilot of the adapted interventions; 17 were randomized to *DKD* (12 in English, 5 in French) and 12 were randomized to *DADA* (with 1 group of xx women conducted in English, and 1 group of xx women conducted in French).

#### Stakeholder Participants and Procedures for Adaptation 

To culturally adapt the interventions, our community partners assisted with participant recruitment, and we conducted virtual focus groups with 68 sexually active participants without HIV or unknown status (per self-report), self-identified African-born Black women aged 18 to 45 and 30 clinicians (See Table 2). Of the non-clinician participants, 61% resided in Massachusetts and 39% in New York, 66% had a degree higher than high school, most (47%) had been in the US for less than 5 years, most (47%) were between the ages of 30 and 39 years. Of the clinician participants, 60% resided in Massachusetts and 40% in New York; 76% identified as Black/African American of which 67% were African-born Black women and 93% were female.

**Table 2:** Stakeholder socio-demographic adaptation

	Client Participants (*n* = 68) *n*(%)	Provider Participants (*n* = 30) *n*(%)
*Current residence*		
Massachusetts	39 (57.4)	18 (60.0)
New York	25 (36.8)	12 (40.0)
Missing	4 (5.9)	
*Gender*		
Male		2 (6.7)
Female	68 (100)	28 (93.3)
*Race/Ethnicity*		
African-born Black	64 (94.1)	20 (66.7)
African American/Black		3 (10.0)
White		3 (10.0)
Asian/Pacific Islander		3 (10.0)
Other		1 (3.3)
Missing	4 (5.9)	
*Age of participants*		
20–29yrs	17 (25.0)	8 (26.7)
30–39yrs	32 (47.1)	10 (33.3)
40–49yrs	10 (14.7)	4 (13.3)
50–59yrs	4 (5.9)	4 (13.3)
60–69yrs	1 (1.5)	2(6.7)
Missing	4 (5.9)	2 (6.7)
*How long have you been in the U.S.*		
Less than 5yrs	32 (47.1)	
5–10yrs	15 (22.1)	
10–15yrs	6 (8.8)	
More than 15yrs	11 (16.2)	
Missing	4 (5.9)	
*Highest level of education*		
Graduate and above	25 (36.8)	
College/University graduate	17 (25.0)	
High school	8 (11.8)	
Some college	3 (4.4)	
Some high school	8 (11.8)	
Technical/Vocation	3 (4.4)	
Missing	4 (5.9)	

During the focus groups, participants were asked to provide perspectives on how to adapt SISTA and S2S for African-born Black women. In alignment with literature [42], greater emphasis was put on considering the cultural belief practices and inclusive-language that would meet the unique needs and challenges of African-born Black women. How the interventions should be facilitated, their length of time, and the content were all adapted to fit the needs of African-born Black women.

#### Description of Culturally Adapted Intervention Content and Structure 

Table 3 shows the original and adapted features of the interventions. The interventions were renamed *DADA* (from SISTA) and *Dada Kwa Dada* (*DKD*; from S2S); “Dada” means “sister” and “kwa” means “to” in Swahili. The renaming of the interventions emerged from focus group discussions, highlighting that having an African-named intervention may foster a stronger cultural connection, enhance community engagement, and increase the relevance and acceptance of the interventions among the target population. The adapted interventions, *DKD* and *DADA*, retain the theoretical basis of Social Cognitive Theory while incorporating elements from the Theory of Gender and Power. Both interventions are peer-led and emphasize empowerment through education and skill-building. In terms of format, *DKD* maintains a one-on-one interaction format with peers, incorporating short videos, didactic information, and a manual within a one-hour session. *DADA* follows a small group format led by two peer facilitators, integrating ethnic storytelling, social support, and interactive learning over two sessions lasting three hours each. The core content of the adapted interventions retains the focus on HIV prevention, including condom use, negotiation skills, and HIV transmission education. However, *DADA* emphasizes ethnic and gender pride through storytelling and incorporates additional elements such as coping skills and behavioral self-management training. Furthermore, both adapted interventions introduce virtual delivery methods, education about PrEP, female and male condom use videos and HIV educational videos featuring African-born Black women, catering to diverse cultural and linguistic needs with the inclusion of a French version. These adaptations enhance accessibility and relevance for the target audience. In addition, each adaptation involved an assessment to better help facilitators tailor the intervention to the women present in each session.

**Table 3:** Intervention descriptors of original (S2S and SISTA) and adapted (DKD and DADA) interventions

	Original Interventions	Adapted Interventions
	S2S“Sister to Sister"	DKD“Dada-kwa-Dada”
Facilitator	Health Professional (nurse, case manager, health educator)	Peer-led (1 facilitator who is African/Black woman)
Format	One-on-one (Individual-level) manualized in-person interaction in English with short video, role plays, didactic information	One-on-one (Individual-level) manualized online interaction in English or French, short video on condom use, didactic information
Length	Brief (20 min)	1 hour
Theoretical basis	Based on Social Cognitive Theory	Based on Social Cognitive Theory
Core content	o Bolster expectations of condom use (e.g., using condoms doesn’t decrease sexual pleasure) o Teach & practice negotiation and refusal skills o Demonstrate condom use o Build self-efficacy for wanting to use condoms	o Risk assessment inventory o HIV education [including HIV transmission, condom use, and PrEP] & negotiation videos [featuring African-born Black women] o Negotiation skills

	SISTA “Sisters Informing Sisters about Topics on AIDS”	DADA
Facilitator	Peer (female)	Peer-led (2 female African facilitators)
Format	Small in-person manualized group session (6–8 peers) in English, ethnic pride poetry, short video, role play, social support, interactive learning	Small group online session (6–8 peers) in English or French, African ethnic story, short video on condom use, social support, manual and handouts, interactive learning
Length	Five 2-hour sessions	Two 3-hour sessions
Theoretical basis	Based on Theory of Gender and Power & Social Cognitive Theory	Based on Theory of Gender and Power & Social Cognitive Theory
Core content	o Inform gender inequality & HIV risk o Promote ethnic & gender pride through African American Poem o Teach women sexual assertion skills o Teach sexual health, anatomy, and communication with partners o Emphasize the importance of the partner’s involvement	o Personal values assessment o African ethnic & gender pride (including African woman’s story) o HIV education including HIV transmission, condom use, and PrEP o Behavioral self-management training o Coping skills

Prior to joining the sessions, *DKD* participants completed a Risk Assessment Inventory before the intervention. This inventory (provided in English and French) aimed to assess the risk of HIV, by evaluating their sexual behaviors, including condomless sex, sex for money or drugs, and sex with high-risk individuals, as well as their experiences with STIs, substance use, and relationship safety. Additionally, the assessment covered concerns about food security and immigration or legal status.

*DADA* participants completed a personal values assessment, which evaluated the definition and discussion of values, the importance of having one’s own set of values, and how these values impact personal decision-making. Participants ranked various values (e.g., hard work, respect for elders, physical activity) from least to most important. Both assessments were used to assist intervention facilitators in tailoring each session to the participants. These personalized approaches ensured that the content and delivery of the interventions were aligned with the specific needs and characteristics of participants.

#### Theatre Testing

 After adaptation, interventions underwent refinement through theater testing, an approach inspired by social marketing to obtain feedback perceptions and gather suggestions for enhancement [33]. In two convened focus groups, participants (research and non-research study team) observed facilitators run through the delivery of the interventions in mock sessions. Subsequently, participants offered feedback on aspects that functioned well and areas requiring further refinement, crucial steps preceding the pilot study.

### Phase 2: Pilot Testing

#### Participants and Procedures

Pilot testing occurred over 3 months in the greater Boston area, MA and New York, NY. Individuals were eligible if they were between 18 and 45 years old, born in an African country, identified as Black or mixed race, without HIV or of unknown status, self-identified as a cisgender woman, reported condomless vaginal or anal sex with one or more men in the last 3 months prior to enrollment, were based in MA or NY, and were fluent in English or French. Participants were recruited through ASC and MAC because recruitment through trusted community partners can play a significant role in mitigating biases and ensuring diverse representation within research studies. These partners had established relationships and credibility within their respective communities, which allowed them to reach our population of interest unencumbered. Their involvement also helped mitigate bias and ensure representation across diverse cultural perspectives and socioeconomic statuses, fostering a more inclusive and accurate approach to participant recruitment and engagement.

In addition to these community partners, our pilot engaged with two clinics (Brigham and Women’s Hospital Gynecology Clinic in MA and the Institute for Family Health in NYC) for referral sites for HIV clinical services including PrEP. After recruitment, participants were randomized to either *DADA* (SISTA) or *DKD* (S2S), stratified by state [43]. We used a blocked 1:1 randomization design with stratification by African region of origin based upon the most common regions of origin of African-born immigrants in the US (West vs. East) and language (English vs. French) [44, 45]. This randomization approach ensured that groups were balanced and comparable by controlling for key demographic and cultural variables. Our approach minimized confounding factors related to regional and linguistic differences, enhancing the internal validity and generalizability of the study findings.

As shown in Table 4, a convenience sample of 29 women (18 English-speaking, 11 French-speaking) participated;17 were randomized to *DKD* (12 in English, 5 in French) and 12 were randomized to *DADA* (with 1 group of six women conducted in English, and 1 group of six women conducted in French). DKD participants had a higher proportion of individuals with household incomes below $50,000 (70.5% vs. 50%). Years in the U.S. varied significantly, with DKD participants more likely to have lived in the U.S. for fewer than five years (41.2% vs. 25%), while DADA participants were predominantly in the 5 to < 10-year range (66.7% vs. 29.4%). Regional birth origins also differed, with DKD participants primarily from Western Africa (76.5%), while DADA participants were more evenly distributed across Western (33.3%), Central (16.7%), and Eastern Africa (50%). Both groups showed limited condom use (DKD: 5.9%; DADA: 0%) and no prior PrEP use, although DKD participants were slightly less familiar with PrEP (70.6% vs. 58.3%). HIV testing rates were similar across groups (DKD: 70.6%; DADA: 66.7%).

**Table 4:** Pilot participant Socio-demographic characteristics and HIV preventative behavior

	DKD “Dada-kwa-Dada” (*n* = 17) M (SD) or % (*n*)	DADA (*n* = 12) M (SD) or % (*n*)
*Age*	34.2 (6.6)	33.2 (5.3)
>High school education	64.7% (11)	75% (9)
Household income (<$50,000)	70.5% (12)	50.0% (6)
*Years in the US*		
Less than 5 years	41.2% (7)	25.0% (3)
5 to < 10 years	29.4% (5)	66.7% (8)
10–15 years	0% (0)	0% (0)
> 15 years	29.4% (5)	8.3% (1)
*Region of birth in Africa*		
Western Africa	76.5% (13)	33.3% (4)
Central Africa	0% (0)	16.7% (2)
Eastern Africa	23.5% (4)	50.0% (6)
*Relationship status*		
Single	31.3% (5)	8.3% (1)
Committed relationship	12.5% (2)	33.3% (4)
Casual relationship	6.3% (1)	0% (0)
Legally married	50.0% (8)	50.0% (6)
Divorced	0% (0)	8.3% (1)
*Intervention language*		
English	70.6% (12)	50.0% (6)
French	29.4% (5)	50.0% (6)
*Location*		
Massachusetts	41.2% (7)	25% (3)
New York	58.8% (10)	75%(9)
Condom use (Always)	5.9% (1)	0% (0)
Never taken PrEP	100% (17)	100% (12)
Never heard of PrEP	70.6% (12)	58.3% (7)
Have tested for HIV before	70.6% (12)	66.7% (8)
*Acceptability of intervention*		
Information quality	75%	90.9%
Delivery quality	70%	100%
Perceived usefulness	93.7%	100%
Overall satisfaction	93.7%	100%

Incentives provided to participants were proportional to the time commitment required for participation to ensure fairness and avoid perceptions of inequity. *DKD* participants received a $50 gift card upon completion of their one-hour session, while *DADA* participants received $125 for completing a two 3-hour session as part of the pilot program this incentive system was designed to reflect the difference in time commitments between the two interventions. All sessions were conducted confidentially, with privacy and confidentiality maintained during virtual sessions by utilizing secure video conferencing platforms with password-protected access. Participants were instructed to join sessions from private locations, and facilitators ensured that no identifying information was shared during discussions. Additionally, participants in the group sessions were explicitly asked not to share any information discussed outside of the group. All data collected during the sessions were securely stored and anonymized to protect participants’ identities.

Additionally, participants were required to complete a baseline survey, undergo a risk inventory assessment, and participate in a post-intervention interview (described below). Participants could refer up to three potential participants to the study; they received $10 for each referral ($30 total in gift cards).

We measured feasibility through administrative data documenting recruitment and retention in the intervention sessions [34–37].

#### Facilitator Training, Supervision and Fidelity

Four facilitators (MPH-level African-born Black female experts) were trained by author LMB on facilitation skills (e.g., engaging participants, dealing with group conflict), by authors BO and KD on HIV/PrEP, and by author GM on intervention manual content. Supervisors [two clinicians (authors BO, KD) and two HIV researchers (authors LMB, NM)] provided guidance about facilitation, subject matter expertise, and group management. Weekly check-ins were conducted with facilitators to discuss their experiences, address challenges encountered during delivery, and provide tailored support. Additional training sessions were held throughout the study to refine facilitation techniques and ensure they aligned with each intervention’s goals.

Two study team members per session (authors NM, KD, LMB, GM) independently rated each session for fidelity to the protocol using standardized forms tailored to the content and goals of the session. For instance, fidelity items included statements such as, “Discussed key facts/statistics on rates of HIV among African-born women” and “Explained PrEP overview” on a 3-item scale of Not at all to Completely. Facilitators completed ratings after each session to record whether or not core session activities were adequately completed on a scale of 1 (*not at all*), 2 (*somewhat*), and 3 (*completely*).

Across session observers, high fidelity (98%) to intervention content was observed, indicating that most content was covered completely. HIV education, prevention strategies, and discussions on PrEP, were comprehensively covered in all sessions, as indicated by 100% completeness. Furthermore, facilitators consistently checked if participants completed the risk assessment inventory (100% completeness) and addressed concerns related to PrEP (74% completeness).

### Assessment

Participants in both interventions completed a baseline survey assessing sociodemographic characteristics (e.g., gender, language), HIV knowledge [46–48] (e.g., Having sex with someone who has HIV is the only way to become HIV positive), HIV prevention behavior (e.g., Have you ever taken PrEP medication, such as Truvada), attitudes (e.g., I could successfully negotiate with my partner to use condom even if he doesn’t want to use one), and sexual activity(e.g., How many sexual partners have you had in the past year?). This manuscript does not analyze baseline survey data (outside of sociodemographic characteristics), as its focus is on the feasibility and accessibility of the interventions. However, the baseline data will serve as a valuable resource for informing a fully powered randomized control trial study by providing insights into participants’ initial knowledge, attitudes, and behaviors related to HIV/PrEP.

Acceptability was assessed using the Information Systems Success Model (ISSM) which was adapted to assess facilitator and participants’ perception of the intervention using four domains (1) information quality, (2) delivery quality, (3) perceived usefulness and (4) overall satisfaction with the intervention using Likert scales [49–53]. Summary scores were derived for each category by calculating the mean of responses in each section and presented in Table 4.

### Post Intervention Interviews and Feedback

Following the pilot, participants were later interviewed for 30 min and surveyed to assess intervention content, acceptability, cultural relevance, and behavioral impact. Interviews were transcribed verbatim, with translation from French to English as necessary. We evaluated the acceptability of the intervention, which refers to the extent to which both those delivering and receiving the intervention perceive it as appropriate [49]. This assessment was conducted through participant online post-session evaluation forms following each session, as well as through interviews or focus groups held immediately after the final intervention session for each participant. The post-session evaluation forms included questions such as, “Overall, how much do you agree with the following statements about the workshop today, on a scale of 1 to 5?“, ranging from 1 (strongly disagree) to 5 (strongly agree). These questions aimed to assess the relevance and usefulness of the information presented, the quality of delivery, the provision of resources, and overall satisfaction. Furthermore, the interviews and focus groups explored participants’ general attitudes toward the program, as well as attitudes regarding program content (e.g., on prevention advocacy), program structure (e.g., number and timing of sessions), and the facilitators.

Two authors (NA, EHF; MPH-level African-born Black scholars) conducted the post-intervention interviews and focus groups. The interviewers are trained in qualitative methods in behavioral science and health sciences research, coupled with prior experience conducting interviews and analyzing qualitative data.

### Data Analysis

For descriptive purposes, we generated means with standard deviations and frequencies as appropriate for all sociodemographic and HIV prevention characteristics. Additional baseline survey data was not analyzed in this manuscript because the focus is on the feasibility and accessibility of the interventions. All analyses were conducted using SAS 9.4 version.

Two independent researchers (NA, EHF) conducted qualitative data analysis using thematic analysis methods. Initially, the data was first assessed through open coding and a descriptive label attached to it [54–56]. Authors (HA, EHF, EDJ, KD, GM) reviewed and defined themes by independently coding four transcripts (two from English and two from French interviews) in a Word document. This initial process resulted in five themes and fourteen sub-themes that captured diverse participant experiences and perspectives. Subsequently, the authors convened to discuss each coded transcript, addressing any ambiguity to ensure consistency and coherence in the thematic structure. Through this iterative process, overlapping codes were merged (e.g., sufficiency of content and useful information were merged as one), unclear codes were clarified, and the final codebook was developed with twelve codes to guide the thematic analysis. To establish consensus on codes and themes, the authors engaged in further discussions. Data from the coded transcripts were then transferred to Excel for inter-rater reliability testing, resulting in an acceptable Cohen’s Kappa of 0.79 given a threshold of 0.70 [57].

All interview transcripts were uploaded to Dedoose, a software platform that facilitates qualitative data management and analysis. Codes were added and applied to sections of text corresponding to identified themes. To address coding disagreements, coders met and discussed code ambiguity which was helpful for understanding codes and refining codes [34, 36, 56].

## Results

### Feasibility

Both interventions had high feasibility: 92% of 12 *DADA* participants and 100% of 17 *DKD* participants attended all intervention sessions. Of the six English-speaking *DADA* participants, five attended and completed the first English session and post-session evaluation, and one missed the second English session due to unexpected events. All six *DADA* French-speaking participants took part in both sessions and completed both evaluations. All *DKD* participants, both French and English speakers, attended all sessions and completed the post-intervention feasibility survey.

The post-pilot interviews did not reveal significant concerns raised by participants regarding the intervention itself. However, several logistical challenges emerged during implementation. Three participants rescheduled their sessions after forgetting about the program, while others faced distractions at work that interfered with their participation. Additionally, a few participants encountered technical difficulties, such as trouble logging into the online platform or using the provided link, which caused delays in joining the virtual sessions. To address these challenges, the research team implemented strategies to enhance feasibility. Research assistants allocated additional time beyond the scheduled intervention period to accommodate late arrivals, ensuring that delays did not overlap with subsequent sessions. To further streamline access, links were sent via text message shortly before the session start time, reducing reliance on email communication. These adjustments minimized disruptions and highlighted the importance of flexible scheduling, proactive communication, and technical support in virtual intervention delivery.

### Acceptability

Post-intervention assessments revealed that 95% of *DADA* and 93% of *DKD* participants found the information quality useful, 82% of *DADA* and 87% of *DKD* participants reported high delivery quality, 100% of both intervention groups perceived the intervention information to be useful, and 100% of both groups also were satisfied with the interventions. On a Likert scale of 1 to 5 with 5 representing high acceptability, DADA participants reported a mean and standard deviation of 4.5 (0.3) and *DKD* had 4.2 (0.3).

Participants qualitatively expressed deep appreciation for both interventions’ comprehensive approach, to not only educate about HIV but also address various aspects of women’s lives. Both interventions received overwhelmingly positive feedback, with *DADA* being particularly endorsed for its group setting, “Group discussion is really important for gaining knowledge about HIV…”. *DADA* participants, both English and French-speaking, praised the sessions for fostering a comfortable and informative environment reminiscent of family discussions, emphasizing the importance of open dialogue on health and sexuality, especially for African immigrant women. Many participants expressed eagerness to recommend the program to others, underscoring its significance in fostering community, knowledge sharing, and self-advocacy among women confronting similar challenges.

**Table:** 

	DADA (Original intervention SISTA)	Dada kwa Dada (Original intervention S2S)
English Speaking Participant	*“I loved DADA. I have never seen this kind of group session on HIV and [other health] topics before. I like that the group was very open and gave a sense of community. It was a good experience for sharing stories with others and learn from other women’s life experiences. Group discussion is really important for gaining knowledge about HIV. Thank you very much for sharing this information with me. This is so important nowadays for African women*,* especially like me who has never gotten sexual health education.”*	*“It was useful because it gave examples and it literally tells you how to approach and how to talk. And also how to take the time. And also understand that it might not happen right there. It’s a process for the person as well. So don’t be mad if the first time you bring it up you don’t get what you want. It’s a process in the person. It’s okay for the person to go and think about it and come back to you. So that’s one thing I think it was important for me to understand that.”*
French Speaking Participant	*“The part that I liked the most or how to maintain*,* talk to your husband*,* talk to your husband. I also really liked that part because as African women*,* it’s not easy for us to face or to talk to our husband. So*,* if others follow the program*,* there’s a lot of advice in here.”*	*“There’s a lack of communication in many couples. Encouraging women to start talking to their partners*,* especially about sex*,* because some women don’t talk about it much*,* even with their partners. And how do you start a conversation? That’s the big problem here*,* so a lot of women don’t talk about it because they don’t know how to start. That was helpful”*

### Cultural Appropriateness

#### Cultural Sensitivity and Addressing Taboo Topics

 Participants consistently highlighted the interventions’ cultural sensitivity, particularly in addressing taboo topics such as sexual practices, partner communication, and HIV-related issues. For many African immigrant women, these subjects are often stigmatized, making open discussion challenging. Participants expressed gratitude for the program’s ability to foster free and open dialogue, breaking down barriers of shame and stigma. For example, one French-speaking participant noted, “Back home, these topics have a lot of stigma. There is a lot of shame.” By creating a safe space for these conversations, the interventions empowered participants to engage with essential yet often stigmatized information.

#### Community and Empowerment

 The interventions were praised for fostering a sense of community and empowerment, which participants identified as particularly relevant for African immigrant women. DADA participants emphasized the importance of group dynamics in building a sense of belonging and mutual support. One participant remarked, “I think DADA is good for African immigrant women, especially because of the feeling of community. Group is important to us.” This sense of community enhanced the intervention’s cultural appropriateness by aligning with values of collective support and connection. By leveraging the cultural importance of communal learning, the interventions created an environment where participants felt motivated to challenge stigma and apply the lessons to their own lives.

#### Cultural Adaptations Language Translation and Storytelling 

The inclusion of French translations was a critical adaptation that ensured accessibility for Francophone participants, many of whom expressed appreciation for receiving information in their preferred language. One French-speaking DKD participant stated, “The fact that the program is in French is going to help African women a lot, and the videos are also really adapted for African women.” This adaptation bridged linguistic barriers and fostered inclusivity, making the intervention feel more tailored to the needs of African immigrant women. Similarly, the use of ethnic storytelling resonated deeply with participants, as it connected the intervention content to their lived experiences. Storytelling served as a culturally familiar method of communication, helping participants relate to the material and understand its relevance. For example, participants noted that the narratives helped them reflect on sensitive issues in their relationships and empowered them to develop negotiation and communication skills. One DKD participant shared, “Especially when you look at the bit of the negotiation skills. You find that most African immigrant women or African women find it very difficult to talk about the most sensitive issues in their relationships. And yet they’re very important.” These adaptations addressed both linguistic and cultural barriers, ensuring that the interventions resonated with participants’ unique experiences.

#### Impact on Stigma, Empowerment, and Health Outcomes 

Many *DADA* participants emphasized the significance of community and empowerment, noting the program’s relevance in enhancing communication skills within a new cultural context. Conversely, *DKD* participants emphasized the importance of negotiation skills when discussing issues with their partners. Across both interventions, participants emphasized the programs’ potential to combat stigma, empower individuals, and improve health outcomes within the African immigrant community. For example, a DKD participant noted, “Basically, we Africans don’t know much about HIV, even if we learned about it in school. Not all African women have been to school. There are many who are not informed, and there are other technologies that have been developed in the intervening years that we didn’t know about.”

**Table:** 

	DADA (Original intervention SISTA)	Dada kwa Dada (Original intervention S2S)
English Speaking Participant	*“I think DADA is good for African immigrant women*,* especially because of the feeling of community.* ***Group is important to us***.*”*	*“Especially when you look at the bit of the negotiation skills. You find that most African immigrant women or African women find it very difficult to talk about the most sensitive issues in their relationships. And yet they’re very important.* ***So the negotiation skill or that section***,*** it’s very important***.*”*
French Speaking Participant	*“****It is very appropriate for African immigrant women***. *I am from West Africa and back home*,* these topics have a lot of stigma. There is a lot of shame.”*	*“Basically*,* we Africans don’t know much about HIV*,* even if we learned about it in school. Not all African women have been to school. There are many who are not informed and there are other technologies that have been developed in the intervening years that we didn’t know about.* ***The fact that the program is in French is going to help African women a lot***,*** and the videos are also really adapted for African women***.*”*

### Increased Knowledge

Participants said that both the DADA and DKD interventions were highly effective in increasing their knowledge, particularly around topics such as PrEP, condom use and broader sexual health practices. Participants expressed gratitude for the opportunity to learn new information and engage in open dialogue, which many noted was a rare opportunity in their cultural context. For example, participants highlighted the importance of advocating for oneself in relationships and expressed appreciation for the insights gained during the sessions. One DADA English-speaking participant shared, “I think African immigrant women don’t really advocate for themselves. And not that they don’t, but in a lot of cases we don’t talk, we don’t share our opinion, we don’t advocate for ourselves in relationships. And yeah, we learned a lot of things about it.”

Similarly, French-speaking participants found the program to be informative and valuable, extending beyond HIV awareness to encompass a broader range of topics. A DKD participants echoed similar sentiments, emphasizing the novelty of learning about PrEP. One participant stated, “No, the pill [PrEP] was new. I didn’t know about it.” Participants contrasted this experience with the fear and misinformation associated with HIV in their home countries, highlighting how the interventions not only filled critical knowledge gaps but also shifted perceptions about HIV prevention and sexual health.

**Table:** 

	DADA (Original intervention SISTA)	Dada kwa Dada (Original intervention S2S)
English Speaking Participant	*I think African immigrant women don’t really advocate for themselves. And not that they don’t but in a lot of cases we don’t talk*,* we don’t share our opinion*,* we don’t advocate for ourselves in relationship. And yeah*, ***we learned a lot of things about it.***	*“PrEP. I had never heard of it before. [the facilitator] did a good job answering all of my PrEP questions.* ***It is helpful to learn that there are so many ways to protect myself now.*** *Back home*,* HIV was so much scarier because you thought you were helpless against it*,* but now with this information*,* I know that you can live with HIV and can also protect yourself from it in many ways.”*
French Speaking Participant	*“Honestly*,* it’s a good program.* ***I really liked it because it allowed me to learn things that I didn’t know***. *The goal of the program was to make us aware of HIV*,* but we learned more than that. It’s a really good program that’s not just based on HIV*,* it brings everything together because we learned a lot. I really liked it.”*	*“No*,* the pill [PrEP] was new.* ***I didn’t know about i****t.”*

### Anticipated Behavior Change

#### Negotiation Skills and Empowerment 

One of the key components of the interventions was the emphasis on negotiation skills, which participants said enabled them to address sensitive topics within their cultural and relational contexts. Participants said that the interventions provided practical tools to help participants initiate these conversations and advocate for their needs. For example, one DADA participant shared how negotiation skills empowered her to discuss HIV testing with her husband before he entered into additional marriages, stating, “…as we’re Muslims, at any time, my husband can take a second wife, a third wife. So, if now, in case it also happens, I suggested that he could get tested before committing to a relationship.” Similarly, participants expressed an increased willingness to negotiate condom use and explore other prevention methods, such as PrEP, despite initial uncertainty or discomfort. One DKD participant noted, “Okay. So definitely this is going to make me use them [condoms] more, like all the time now, and be more cautious about it.”

#### Group Discussions and Peer Support

 Group discussions (DADA) served as a key intervention element, leveraging the collective setting to foster shared experiences and mutual support, compared to a one-on-one setting (DKD). For many DADA participants, the sense of community and mutual understanding fostered was transformative. One DADA participant remarked, “I like that the group was very open and gave a sense of community.” The group dynamic not only facilitated skill-building but also normalized conversations about sexual health, reducing feelings of shame or isolation. Participants also highlighted the importance of hearing diverse perspectives within the group, which helped them contextualize their own experiences and challenges, “It was a good experience for sharing stories with others and learn from other women’s life experiences.” For example, discussions about cultural stigma surrounding HIV and sexual health allowed participants to collectively address these barriers and develop strategies to overcome them. This peer support reinforced the interventions’ messages and encouraged participants to apply the skills they learned in their own lives. The group format also leveraged shared cultural experiences, such as migration-related experiences and collective attitudes toward gender and sexual health, creating a space where participants could relate to one another and feel empowered to challenge societal norms.

#### Acknowledging Challenges and Commitment to Change

 Despite the effectiveness of negotiation skills and group discussions, some participants acknowledged the difficulty of applying these lessons in their everyday lives due to cultural and relational barriers. One participant expressed this challenge poignantly, stating, “I strongly believe I will apply DADA to my life but it will not be easy. These topics are hard to talk about. Sometimes, it’s so hard to get someone to understand you, especially when you are from Africa and someone is telling you, nah, you don’t know anything.” This skepticism echoed concerns about potential resistance from family members or peers, an uncertainty about whether their efforts would be supported or understood.

**Table:** 

	DADA (Original intervention SISTA)	Dada kwa Dada (Original intervention S2S)
English Speaking Participant	*“I strongly believe I will apply DADA to my life but it will not be easy. These topics are hard to talk about. Sometimes*,* it’s so hard to get someone to understand you*,* especially when you are from Africa and someone is telling you*,* nah*,* you don’t know anything”*	*“Okay. So definitely this is going to make me use them [condoms] more*,* like all the time now*,* and be more cautious about it.”*
French Speaking Participant	*“…as we’re Muslims*,* at any time*,* my husband can take a second wife*,* a third wife. So*,* if now*,* in case it also happens*,* I suggested that he could get tested before committing to a relationship”*	*“The program allowed me to have confidence in myself first*,* to plan my sexual life and to plan it with my partner.”*

## Discussion

This study marks the first attempt, to our knowledge, to culturally and linguistically adapt HIV prevention interventions for African-born Black women living in the US. Our culturally adapted interventions, *DADA* and *DKD*, demonstrated high intervention acceptability and feasibility, with preliminary outcomes aligning with our hypotheses. Our results further suggested that *DADA*, a group-level intervention that tackles gender inequality and fosters a sense of community, was especially acceptable in helping participants to feel comfortable discussing sexual health issues. The group format of DADA played a critical role in fostering interaction, mutual support, and peer learning, which were instrumental in empowering participants to overcome cultural stigma and navigate sensitive topics. Participants highlighted the value of connecting with other women who shared similar cultural backgrounds and challenges, with participants noting, the value of the group-based intervention. This sense of belonging facilitated open dialogue and collective problem-solving, enabling participants to share strategies for negotiating HIV testing and advocating for safer sexual practices. These findings highlight the importance of group-based interventions in enhancing knowledge acquisition, building confidence, and promoting behavior change among African-born Black women as is supported by literature [19].

In addition, findings aligns with recent literature emphasizing the significance of HIV interventions addressing gender inequality and emphasizing the importance of community support in promoting sexual health awareness and behavior change among diverse populations [14]. Participants reported feeling more empowered to negotiate condom use and discuss PrEP with their partners, outcomes linked to intervention components such as role-playing and gender equity discussions. For instance, one participant appreciated learning how to approach sensitive conversations with patience and understanding, while another noted increased confidence in using condoms consistently. These skillsets were enhanced by encouraging the women to roleplay during the virtual sessions to help them practice initiating conversations about condom use and PrEP in supportive and realistic setting. In addition, intervention resources included videos where they saw other African-born Black women navigate these discussions with their partners, providing relatable examples and reinforcing key communication strategies. These culturally tailored strategies addressed deeply rooted cultural norms and stigma, which often discourage open conversations about sexual health, and provided participants with tools to overcome these barriers. Overall, participants believed that the interventions addressed communication challenges related to gender inequality.

Both interventions led to increased knowledge on PrEP use, addressing the limited awareness among African-born Black individuals in the US. These findings are based on a small, convenience sample of African-born women primarily living in Massachusetts and New York, highlighting the importance of such interventions, in improving understanding [12, 58]. Results also indicated that participants not only received useful information but also acquired tools to make proactive behavior changes. After the *DADA* intervention, participants reported initiating discussions with their partners about HIV testing and the importance of consistent condom use to prevent sexually transmitted illnesses. *DKD* participants reported gaining confidence and acquiring skills to initiate behavior change, such as discussing sexual health matters with their partners, advocating for safer sexual practices, and making informed decisions about HIV prevention methods. These results are in alignment with what we have seen among Black/African American women with SISTA and S2S [28, 29].

Our findings also align with the broader context of HIV prevention challenges faced by African-born Black women in the U.S., who often encounter stigma, limited access to culturally relevant information, and insufficient awareness of prevention tools like PrEP [1, 2, 12–18, 20, 21]. The interventions addressed these barriers by creating safe spaces for open dialogue, equipping participants with practical negotiation skills, and tailoring content to reflect their cultural and linguistic needs. For instance, participants reported increased confidence in discussing HIV testing and condom use with their partners—critical steps in overcoming stigma and fostering proactive behavior change. The inclusion of ethnic storytelling and language translation further ensured that the interventions resonated with participants’ diverse cultural backgrounds, addressing the lack of culturally relevant information that has historically hindered HIV prevention efforts in this population. By demonstrating the effectiveness of culturally tailored, community-based approaches, these findings contribute to the growing body of evidence supporting strategies that reduce stigma, improve knowledge, and empower African-born Black women to take control of their sexual health. Additionally, the interventions addressed migration-specific challenges, such as linguistic barriers and stigma tied to adjusting to a new cultural environment, which are often overlooked in broader HIV prevention efforts. This nuanced approach ensured that the interventions were not only effective but also responsive to the unique needs of African-born Black women.

In sum, our findings demonstrate that the core structure and components of the intervention are well-received by African-born Black women, suggesting that similar positive attitudes can be expected among other African-born Black women. However, this pilot study has a few limitations; first it is limited by the small convenience sample and thus caution is advised when generalizing the findings. While recruitment through community partners did enhance representation, relying on a convenience sample could have introduced selection biases and limit the study’s external validity. The findings may not accurately represent broader populations or capture the full diversity of perspectives. Future research will aim for larger, more randomized samples to validate the results and ensure that insights are applicable across different contexts and populations. Secondly, the differences in regional birth origins between DKD and DADA participants can impact the cultural applicability of the interventions by shaping how shared or differing cultural norms, and experiences influenced their reception and outcomes. The DKD group’s predominantly Western African cohort may limit its generalizability, whereas the DADA group’s diverse representation across Western, Central, and Eastern Africa enhances its potential applicability to broader African immigrant populations. Another limitation of this study is that it was conducted in Massachusetts and New York, two urban areas with relatively high concentrations of African immigrant communities and access to healthcare resources, which may not reflect the experiences of participants recruited from other states with differing demographics, healthcare infrastructure, or community support systems. Lastly, a notable limitation of conducting these interventions virtually is the reliance on stable internet access and familiarity with digital platforms, which may exclude individuals with limited technological resources or digital literacy. Furthermore, the generalizability of the intervention’s findings may be limited, as the experiences of participants engaging in virtual sessions may not fully reflect those of individuals who prefer face-to-face interactions.

A fully powered comparative effectiveness trial is needed to assess the relative impact of both interventions on increased condom use and uptake of PrEP. Should the intervention prove effective, scaling up could be facilitated through community-based clinics and organizations.
